# EHBMT, a method for visualizing tumor evolution, identifies a surge in gastric cancer with hybrid epithelial–mesenchymal phenotypes due to an inflammatory microenvironment

**DOI:** 10.1038/s12276-025-01570-6

**Published:** 2025-11-03

**Authors:** Dandan Li, Zeng Zhou, Yuanjian Hui, Hedong Yu, Tao Ren, Lantian Zhai, Xinqi Li, Lin Yuan, Lingyun Xia, Weidong Leng, Shanshan Qin

**Affiliations:** 1 Department of Stomatology, Taihe Hospital and Hubei Key Laboratory of Embryonic Stem Cell Research, School of Basic Medical Sciences, Hubei University of Medicine, Shiya, People’s Republic of China; 2 Laboratory of Tumor Biology, Academy of Bio-Medicine Research, Hubei University of Medicine, Shiyan, People’s Republic of China; 3 Hubei Provincial Clinical Research Center for Umbilical Cord Blood Hematopoietic Stem Cells, Taihe Hospital, Hubei University of Medicine, Shiyan, People’s Republic of China

**Keywords:** Chronic inflammation, Translational research, Epithelial-mesenchymal transition, Gastric cancer, Predictive medicine

## Abstract

A method for analyzing tumor evolution based on bulk RNA-sequencing data has not been reported yet. The epithelial–mesenchymal transition (EMT) is an evolutionarily conserved cellular program with high heterogeneity and plasticity. In this study, we proposed an EMT heterogeneity-based molecular typing (EHBMT) method to visualize cancer evolution and guide personalized medicine. Multiplex immunohistochemical assay and single-cell analysis were performed to confirm the feasibility of this method. EHBMT divided gastric (cancer) tissues into an epithelial phenotype cluster (EPC), hybrid epithelial–mesenchymal phenotype cluster (HPC) and mesenchymal phenotype cluster (MPC). Patients with gastric cancer with different EHBMT subtypes possessed distinct clinical features, molecular characteristics and prognostic outcomes. Furthermore, the proliferation ability of EPC, HPC and MPC subtypes decreases sequentially. Gene Ontology/Kyoto Encyclopedia of Genes and Genomes analysis showed that HPC subtypes are associated with inflammation and immune activation. More importantly, EHBMT discovered a sharp increase in the proportion of the HPC subtype during gastric cancer evolution. Traceability analysis indicated that the surge in HPC in gastric cancer was due to the transition from approximately 70–80% of normal EPC cases to cancerous HPC/MPC cases. In addition, the inflammatory factor IL-1β, highly expressed epithelial cells in the HPC subtype, should be a key driver for the decrease of epithelial cells by inducing EMT signaling. In conclusion, EHBMT is a novel method for visualizing cancer evolution using bulk transcriptomics. Gastric carcinogenesis is accompanied by a sharp increase in the proportion of HPC due to the abnormal EMT signaling pathway driven by an inflammatory microenvironment.

## Highlights

- We herein provided an epithelial–mesenchymal transition heterogeneity-based molecular typing method (EHBMT), which can be applied in normal and cancerous tissues to visualize cancer evolution and development and guide personalized medicine.
- EHBMT divided gastric (cancer) tissues into three subtypes, including an epithelial phenotype cluster (EPC), hybrid epithelial–mesenchymal phenotype cluster (HPC) and mesenchymal phenotype cluster (MPC). Different EHBMT subtypes possess distinct proliferation abilities, molecular characteristics and clinical outcomes in gastric cancer (GC).
- GC evolution is accompanied by a steep rise in proportion of the hybrid epithelial–mesenchymal transition subtype with significant inflammatory and immune activation features.
- During GC evolution, about 70–80% of normal EPC cases were transited into cancerous HPC/MPC cases. An inflammatory microenvironment, especially IL-1β, should be a key driver for the transition from the normal EPC subtype to the cancerous HPC subtype in GC.

## New method visualizes tumor evolution through EMT heterogeneity

Cancer development is a complex process with many differences between patients and even within a single tumor. Here researchers focused on a process called the epithelial–mesenchymal transition (EMT), which helps cancer cells spread. They developed a method to classify stomach cancer based on EMT differences using data from various sources, including RNA sequencing. This method divides cancer into three types: epithelial, hybrid and mesenchymal, each with different characteristics and outcomes. The study found that the hybrid type is more common in cancerous tissues than in normal ones. This suggests that changes in EMT are linked to cancer progression. The researchers used advanced techniques such as single-cell analysis and immunohistochemistry (a method to visualize proteins in tissues) to confirm their findings. They concluded that understanding the EMT can help predict cancer behavior and guide treatment decisions.

This summary was initially drafted using artificial intelligence, then revised and fact-checked by the author.

## Introduction

Malignant tumorigenesis, also known as carcinogenesis, is a complex multistep biological process with high heterogeneity^1^. On the one hand, there are significant histological, transcriptomic and genomic differences among patients with cancer, known as interpatient heterogeneity. On the other hand, with the rise of single-cell sequencing technology, more studies have noticed that there is also a high degree of heterogeneity in the cell population within tumors, known as intrapatient heterogeneity^2–4^. These high interpatient and intratumor heterogeneities ultimately determine the clinical presentation, response to treatment and prognosis differences^5^. Therefore, personalized medicine is crucial in tumor treatment.

Cancer origin and progression are spatial processes that involve the breakdown of normal tissue organization, invasion and metastasis^6^. Cancer evolution and development (Cancer Evo–Dev) is the process of observing the occurrence, progression and metastasis of cancer from a temporal perspective, essentially explaining the spatial and temporal evolution of heterogeneous cancer cell populations^7^. Tumors are essentially a complex dynamic ecological system where heterogeneity is not only a substrate for evolution, but can also promote, or even be a requirement for, continued tumor development and progression^8^. According to recent advances regarding Cancer Evo–Dev, gene mutations, chronic inflammation, uncontrolled proliferation, immune escape and the tumor microenvironment have been identified as driving factors for cancer. The updating and development of the Cancer Evo–Dev theory is crucial for providing new ideas, strategies and technologies for cancer prevention and treatment.

Precision medicine requires a better understanding of Cancer Evo–Dev, as well as the heterogeneous population of tumor cells that spans space and time. Molecular typing has been widely used in personalized medicine for patients with cancer-related disease. The epithelial–mesenchymal transition (EMT) is essentially an evolutionarily conserved program of cellular plasticity that controls the state of cells along the epithelial–mesenchymal axis, conferring EMT plasticity to epithelial cells^9–11^. The EMT process endows tightly anchored epithelial cells with invasive ability by converting them into motile mesenchymal cells, and is therefore considered a crucial step in the early stage of cancer metastasis^12^. Accordingly, increasing studies have confirmed that the EMT program is important for tumorigenesis and cancer metastasis.

EMT is not a binary process but contains a heterogeneous and dynamic disposition with different intermediaries (also known as hybrid epithelial–mesenchymal (E/M) state) or partial EMT meta-states^13^. The p-EMT state, also known as the hybrid epithelial–mesenchymal state (hybrid E/M state or hybrid state) or intermediary state, is located in the intermediate stage between epithelial and mesenchymal states^14^. In other words, EMT is not a simple binary process but actually contains different transition states, including the epithelial state, the mesenchymal state and diverse hybrid E/M states^15–17^.

The EMT process has high heterogeneity and plasticity, and tumor heterogeneity should be partly attributed to the EMT plasticity of tumor cells^18^. In addition, EMT is an evolutionarily conserved cellular program that exists in both normal tissues and tumor tissues. In this case, we can distinguish the high heterogeneity of cancer by molecular typing according to their differences in EMT heterogeneity and plasticity. For example, Cristescu and colleagues have divided patients with gastric cancer (GC) into four subtypes, including microsatellitein stability (MSS)/TP53^−^, MSS/TP53^+^, microsatellite instability (MSI) and MSS/EMT^19^. Likewise, emerging single-cell RNA-sequencing (RNA-seq) studies have confirmed the dynamically configurable heterogeneity and plasticity of EMT^20–22^. In this study, we proposed an EMT heterogeneity-based molecular typing method (EHBMT), which can be applied to both normal and cancerous tissues to guide personalized medicine. We have demonstrated the feasibility of this method using stomach cancer as an example, and validated its reliability through single-cell analysis and multiplex immunohistochemistry (mIHC) experiments.

## Materials and methods

### Data acquisition

The RNA-seq data and clinical information of the Cancer Genome Atlas (TCGA) stomach cancer cohort (TCGA_STAD) was downloaded from the cBiopPortal web server. The transcriptome data of the GSE62254 dataset was downloaded from the Gene Expression Omnibus (GEO) in the NCBI web server. The clinical information of the Asian Cancer Research Group (ACRG) cohort (GSE62254) was downloaded from the supplementary data of ref. ^19^. The transcriptome data of the normal stomach tissues from the GTEx cohort was obtained as we previously described^23^. The transcriptome data of the GSE122401 and the GSE179252 cohorts was obtained as we previously described^24,25^. For the GSE122401 cohort, the normalized gene expression data using the relative standard error of the mean value in the GC cohort (GSE122401) was directly downloaded from the GEO dataset^26^. For the GSE179252 cohort, the gene expression data (count value) were normalized to the fragments per kilobase of exon model per million mapped fragments value.

### DEG analysis

The significant differentially expressed genes (DEGs) of each EMT subtype were identified using the DEseq2 R package. The differentially expressed protein-coding genes with *P* values less than 0.0001 are displayed in the volcano plot. Among them, the significantly upregulated protein-coding genes were collected for Gene Ontology (GO)/Kyoto Encyclopedia of Genes and Genomes (KEGG) analysis. The top 100 significantly upregulated genes are displayed in the heat map (Fig. 6a–c).

### Molecular typing analysis

Our previous review provided a comprehensive overview of the plasticity and heterogeneity of EMT signaling and proposed that EMT plasticity can lead to heterogeneity in GC^18^. On the basis of this, we propose a EMT heterogeneity-based molecular typing method. The unsupervised clustering analysis was performed based on the expression pattern of classic EMT-related biomarkers. The representative EMT signaling-related biomarkers, including epithelial signature genes (KRT18, KRT19, CDH1, EPCAM, F11R, EARP1, ESRP2, CLDN1, CLDN4, CLDN7, S100A14, PRSS8, PRSS22, ST14, ZNF165, C1ORF116, KDF1, CRB3, ELF3, HNF4A, OVOL1 and OVOL2) and mesenchymal signature genes (ZEB1, ZEB2, SNAI1, SNAI2, TWIST1, TWIST2, VIM, TGFB1. TGFB2. TGFB3, FN1, SPARC, COL1A1, COL1A2, MMP2 and TCF4) were based on our work and previous reviews. Clustering was performed on individual datasets and the samples were further classified into three subgroups according to the unsupervised cluster analysis using the ComplexHeatmap package. The clustering method was complete linkage, and the distance measurement method was calculated using the Pearson test. Samples were categorized into three subgroups (epithelial, hybrid epithelial–mesenchymal and mesenchymal) according to their distinct EMT signatures.

The epithelial subgroup samples were labeled with ‘epi-high’ (or mesenchymal-low), the hybrid epithelial–mesenchymal subgroup samples were labeled with ‘epi-medium’ (or mesenchymal-medium) and the mesenchymal subgroup samples were labeled with ‘epi-low’ (or mesenchymal-high). The xCell deconvolution analysis can evaluate the epithelial score of per sample based on the bulk transcriptome data. The Estimation deconvolution analysis can evaluate the stromal score in a sample based on the bulk transcriptome data. To verify the classification accuracy of the EHBMT method, we performed a deconvolution analysis based on xCell and Estimate algorithms on the transcriptome of each cancer cohort to evaluate the degree of epithelial and stromal scores of all samples in each cohort. For example, we defined samples that were classified into the epithelial group by epi-high labeled by the xCell algorithm and mesenchymal-low labeled by the Estimate algorithm.

### Clinical GC samples

Tissue samples (GC tissues and adjacent mucosa) were obtained from postoperative resection samples of patients with GC (*n* = 35) admitted to Taihe Hospital affiliated with Hubei Medical University. The study protocol was approved by the Human Research Ethics Committee of Hubei University of Medicine (no. 2024-TH-021). The procedures were in accordance with the Helsinki Declaration of 1975. Written informed consent was obtained from all patients. Tissue samples were immediately frozen in liquid nitrogen after resection and stored at −80 °C until use. All samples were pathologically confirmed.

### Single-cell analysis

The single-cell RNA-seq (GSE183904) dataset of GC samples containing 31 primary gastric tumor samples representing clinical stages I–IV and histological subtypes was available in the GEO repository^27^. We downloaded the original single-cell RNA-seq data and conducted re-analysis as previously described^28^. Detailed information of the single-cell analysis is given below. The R package Seurat (version 4.1.0) was selected for single-cell analysis. Briefly, once the quality control steps were completed, data normalization and scaling were conducted using the Seurat R package. The 10× data matrices were imported into Seurat V5.1 R package to perform data filtration, sample integration, gene normalization, dimension reduction and data visualization. Cells with feature counts (<200 or >8,000) and a high percent of mitochondrial genes (>20%) were removed. Dimension reduction was done by the Seurat ‘RunPCA’ function. Then, Uniform Manifold Approximation and Projection for Dimension Reduction (UMAP) was used to visualize single-cell clusters by graph-based clustering the cells, employing the top 20 principle components with the largest variance (at a resolution of 0.2 for all the merged samples). The clustering of different cell types was performed by the corresponding classical biomarkers^28^. We characterized the identities of cell types of these groups based on the expression of known markers: CD3D (T cell), TNFRSF17 (plasma B), CDH1 (epithelial), CD163 (macrophage), PECAM1 (endothelial), FN1 (fibroblast), MS4A1 (B cell), KIT (mast). We collectively refer to endothelial cells and stromal cells as mesenchymal or stromal cells. The epithelial-to-mesenchymal ratio (E/M ratio) was used to reflect the progression of EMT in per sample. The E/M ratio was calculated as the number of epithelial cells divided by the sum of the numbers of epithelial and mesenchymal cells.

### mIHC assay

The mIHC assay was conducted using the mIF kit (Servicebio Technology) according to the manufacturer’s protocol. The four-labeling immunofluorescence utilizes tyrosine signal amplification (TSA) technology to simultaneously label four proteins on the same slice. Most of the steps for mIHC are the same as those for IHC. The minor difference is that during the mIHC experiment, TSA treatment and microwave elution are required after incubating the first and second antibodies. The TSA treatment causes fluorescently labeled tyrosine to covalently bind to the target protein tyrosine residues in the presence of HRP and hydrogen peroxide. Since the noncovalently bound first and second antibodies are broken down and washed away by microwave treatment, the covalently bound fluorescent tyrosine remains attached to the target protein. In this way, when detecting the second target, it is equivalent to a new round of labeling and there is no need to consider whether the second round of antibody will have a crossreaction with the first round of antibody. By repeating the immunolabeling process multiple times, different fluorescent tyrosine can be used to achieve dual or multiple fluorescent staining. The primary antibodies and corresponding TSA types used in this study were as follows: E-cadherin (GB12083, TSA-440, Servicebio), VIM (GB121308, TSA-546, Servicebio), Ki-67 (GB151499, TSA-488, Servicebio) and IL-1β (GB12115, TSA-647, Servicebio). Scores for staining intensity: 0, no staining; 1, weak staining; 2, medium staining; and 3, strong staining. Scores for the percentage of positive cells: 0, ~0–5% positive cells; 1, ~6–25% positive cells; 2, ~26–50% positive cells, 3, ~51–75% positive cells; and 4, ~76–100% positive cells. The protein expression level (IHC score) is equal to the product of the staining intensity score and the positive cell ratio score.

### Cell culture

The normal gastric epithelial cell line GES-1 was purchased from the Shanghai Cell Bank. The cells were cultured in DMEM medium containing 10% FBS at 37 °C in 5% CO_2_. For IL-1β protein treatment, 2 ng/ml exogenous recombinant human IL-1β protein (HZ-1164, Proteintech) or negative control (PBS) was added in the medium for co-incubation for 72 h. Then, cells were collected for RNA-seq, immunofluorescence and RT–qPCR assays.

### Immunofluorescence assay

The GES-1 cells were seeded in 10-mm confocal dishes with a density of 1 × 10^5^/well. The rabbit Vimentin antibody (22031-1-AP, Proteintech) and the rabbit E-cad antibody (20874-1-AP, Proteintech) were used as the primary antibodies and the Abflo 594-conjugated goat anti-rabbit IgG (AS039, Abcolonal) was used as the secondary antibody. The results were observed using a laser scanning confocal microscope (Olympus FV3000RS). The experiment was independently repeated three times.

### RNA-seq and RT–qPCR

Total RNA was extracted using Trizol reagent (Invitrogen) according to the manufacturer’s instructions. For RNA-seq, a total amount of 1.5 µg RNA per sample was used as input material for the RNA sample preparations. The whole process of library construction and sequencing was performed at Shanghai Lifegenes Technology Co. Ltd. For qPCR analysis, GAPDH was used as an internal control. Each gene was run in triplicate. Relative fold changes in gene expression were calculated using the comparative ^ΔΔ^*Ct* method. The following primers were used: Vimentin-qF: GGGAGAAATTGCAGGAGGAG, Vimentin-qR: AGGTCAAGACGTGCCAGAGAC; E-cadherin-qF: GAAAGCGGCTGATACTGACC, E-cadherin-qR: CGTACATGTCAGCCGCTTC; TWIST2-qF: CAAGGCATGGCATGGCTTAC; TWIST2-qR: TGGGTGGACGTGTAGAGTCC; GAPDH-qF: TCACCAGGGCTGCTTTTA, GAPDH-qR: AAGGTCATCCCTGAGCTGAA; EpCAM-qF: CGCAGCTCAGGAAGAATGTG, EpCAM-qR: TGAAGTACACTGCCATTGACG; TGFB1-qF: AACCCACAACGAAATCTATG-3, TGFB1-qR CTTTTAACTTGAGCCTCAGC-3; NFKB2-qF: CCATGACAGCAAATCTCC, NFKB2-qR: TAAACTTCATCTCCACCCC; RELB-qF: CAATCCCAACCAGGATGTCT, RELB-qR: AGCCATGTCCCTTTTCCTCT.

### Statistical analysis

For gene expression analysis of different subtypes of GC, the *P* values were estimated using Mann–Whitney nonparametric test. Survival curves were calculated using the Kaplan–Meier method and differences between the curves were analyzed using the log-rank test. The gene expression correlation analysis was done using the Pearson test. The chi square test was used to analyze the significant differences in the proportion of different EMT subtypes in normal and cancer tissues, as well as the correlation between EMT subtypes and clinical characteristics of patients. For the other experiments, unpaired *t*-tests were used. All tests with *P* values less than 0.05 were considered statistically significant.

## Results

### The overall framework and technical roadmap of this study

**Fig. 1:**
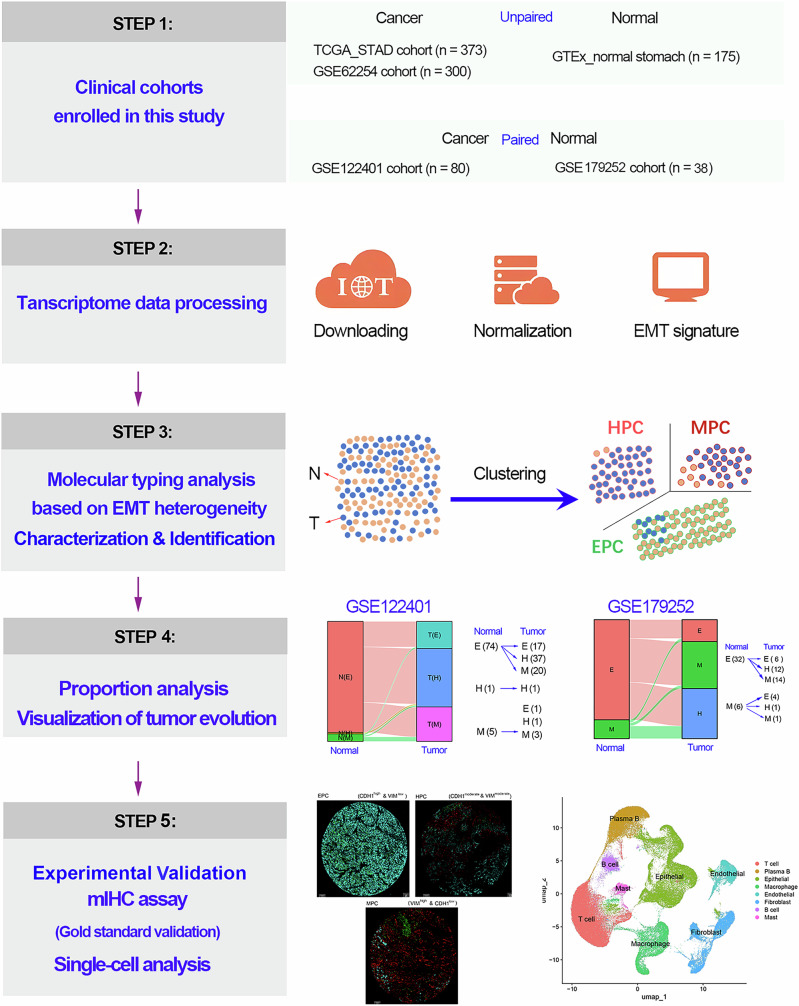
Study design and overview. This study includes an unpaired GC cohort (cancer: GSE62254, TCGA_STAD; normal: GTEx_stomach) and a paired GC cohort (GSE122401, *N* = 80). After transcriptome data processing, the normalized expression of well-known epithelial and mesenchymal biomarkers was applied to evaluated the degree of the EMT progression of each sample. Meanwhile, according to the EMT heterogeneity differences, samples were clustered into different EMT subtypes using complete linkage method. Then, we analyzed the clinical features, prognostic status, molecular characteristics and proportion of different EMT subtypes. Finally, we validated the above molecular typing results using the mIHC method in our own paired GC cohort (*N* = 35).

As previously reported, the EMT process exhibits high heterogeneity and plasticity, which should be one of the reasons for tumor heterogeneity^18^. On the basis of this, we herein proposed a workflow for EHBMT by molecular typing GC tissues and/or normal gastric tissues according to their EMT heterogeneity using bulk transcriptome data (Fig. 1). Given that there are significant regional differences in the incidence and mortality rates of GC, this study included the bulk transcriptome data of the TCGA-STAD cohort (*n* = 373), the ACRG cohort (GSE62254, *n* = 300) and the normal gastric tissue cohort in the GTEx database (GTEx_stomach, *n* = 175). In addition, our study also included the bulk RNA-seq data of the GC cohort (GSE122401 and GSE179252 cohorts) containing paired cancerous and adjacent tissues to validate the feasibility of the EHBMT method. The EMT-related biomarkers, including classic epithelial biomarkers and mesenchymal biomarkers, were selected based on our previous literature review^18^. The EHBMT method was conducted based on the expression signature of these EMT-related biomarker genes in both normal and tumor tissues (Fig. 1). In general, the EHBMT method can divide tissue cohort into three subtypes: a epithelial phenotype cluster (EPC), a hybrid epithelial–mesenchymal phenotype cluster (HPC) and a mesenchymal phenotype cluster (MPC), based on their EMT signature features. Finally, we analyzed the molecular characteristics as well as the proportional changes of different EHBMT subclusters to visualize GC evolution. We further validated the feasibility of the EHBMT method using single-cell analysis and mIHC experiments.

### Molecular classification of GC using EHBMT

**Fig. 2:**
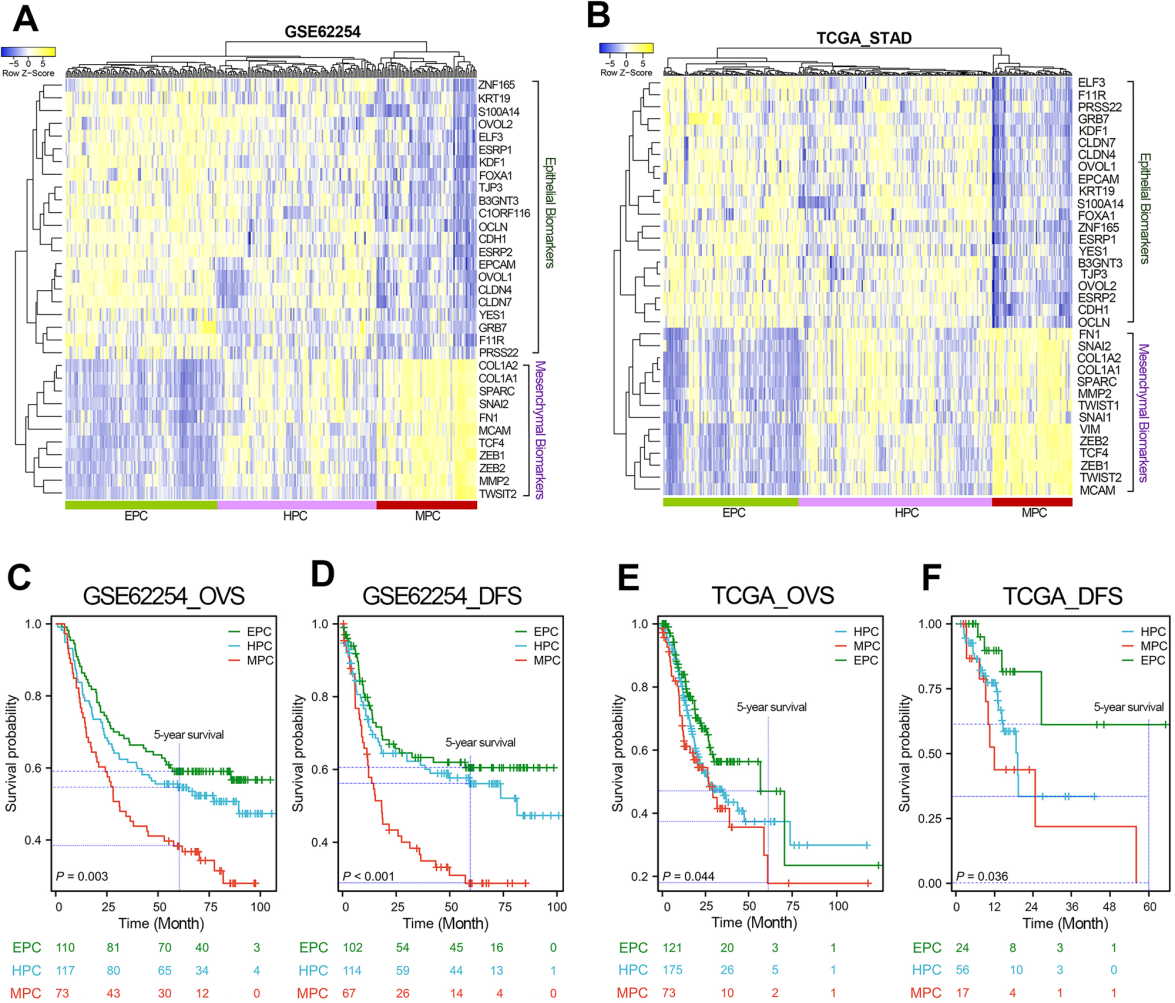
Molecular typing analysis based on EMT heterogeneity in the two independent GC cohorts. **a**, **b**, The molecular typing studies were performed based on the expression signature of classic EMT biomarkers in the two independent GC cohorts (GSE62254 (**a**) and TCGA_STAD (b)). According to the molecular typing analysis, patients with GC from the GSE62254 cohort could be divided into three subtypes as follows: EPC (*n* = 110), HPC (*n* = 117) and MPC (*n* = 73). Patients with GC from the TCGA_STAD cohort could be divided into three subtypes as follow: EPC (*n* = 123), HPC (*n* = 177) and MPC (*n* = 73). **c**, **d**, Overall (**c**) and disease-free (**d**) survival analyses were performed in patients with GC with different EMT subtypes in the GSE62254 cohort. EMT progression was clinically associated with poor prognosis in this cohort. **e**, **f**, Overall (**e**) and disease-free (**f**) survival analyses were performed in patients with GC with different EMT subtypes in the TCGA_STAD cohort. EMT progression was clinically associated with poor prognosis in this cohort.

Based on the expression pattern of classic EMT-related biomarker genes, EHBMT was applied in patients with GC from two independent GC cohorts (GSE62254 and TCGA_STAD). According to Fig. 2a, b, EHBMT can divide patients with GC from the GSE62254 (ACRG) cohort or the TCGA_STAD cohort into three subtypes: EPC, HPC and MPC. The GSE62254 cohort contained 110 patients with the EPC subtype (36.7%), 117 patients with the HPC subtype (39%) and 73 patients with the MPC subtype (24.3%). The TCGA_STAD cohort includes 123 patients with the EPC subtype (33.0%), 177 patients with the HPC subtype (47.4%) and 73 patients with the MPC subtype (19.6%). The detailed clustering information per-GC sample is provided in Supplementary Tables 1 and 2. Next, we conducted survival analysis among patients with GC with different EHBMT subclusters. The prognostic analysis of the two independent GC cohorts both showed that patients with GC in EPC possessed the longest overall and disease-free survival time, while the MPC patient subtype had the shortest overall and disease-free survival time (Fig. 2c–f). In the GSE62254 cohort, the 5-year overall survival rates for EPC, HPC and MPC patient subtypes were 59.1%, 54.5% and 38.4%, respectively, and the 5-year disease-free survival rates were 60.6%, 56.1% and 29.0%, respectively. In the TCGA_STAD cohort, the 5-year overall survival rates for EPC, HPC and MPC patient subtypes were 47.1%, 37.3% and 18.0%, respectively, and the 5-year disease-free survival rates were 61.4%, 33.3%, and 0.0%, respectively. These results suggest that the progression of the EMT program is closely related to poor prognosis in GC.

### Molecular subclusters are associated with clinical characteristics

Next, we analyzed the clinical correlation between different EHBMT subclusters of patients with GC and their pathological characteristics in the GSE62254 and TCGA_STAD cohorts (Tables 1 and 2). Clinical analysis showed that EHBMT subclusters were significantly correlated with histological type, T stage and pathological stage of patients with GC from the two independent GC cohorts. Specifically, the EPC subtype tends to be intestinal GC with shallow infiltration and good prognosis, while the MPC subtype tends to be diffuse GC with deep infiltration and poor prognosis (Tables 1 and 2). In addition, in the GSE62254 cohort, patients in the MPC subtype were more prone to lymph node metastasis (*P* = 0.033) and lymphovascular invasion (*P* = 0.043). In the TCGA_STAD cohort, patients in the MPC subtype typically had poor differentiation (*P* = 0.000) and it was more likely to occur in Asians (*P* = 0.019). The clinical and prognostic analysis results suggest that EHBMT method is reasonable and acceptable as it can establish significant differences in the diagnosis and prognosis of GC.

**Table 1:** The clinical correlation analysis between patient characteristics and different molecular subtypes in the GSE62254 cohort.

Characteristics	EPC	HPC	MPC	*P* value	χ^2^/*t*
*N*	110 (36.7%)	117 (39.0%)	73 (24.3%)		
**Sex**					
Female	37 (33.6%)	39 (33.3%)	25 (34.2%)	0.992	0.017
Male	73 (66.4%)	78 (66.7%)	48 (65.8%)		
**Location of tumor**					
Antrum	64 (58.2%)	61 (52.1%)	30 (41.1%)	0.058	12.203
Body	30 (27.3%)	47 (40.2%)	30 (41.1%)		
Cardia, GE junction	15 (13.6%)	7 (6.0%)	10 (13.7%)		
Whole, multicentric	1 (0.9%)	2 (1.7%)	3 (4.1%)		
**Lauren type**					
Intestinal	74 (67.3%)	51 (43.6%)	21 (28.8%)	0.000	35.229
Diffuse	26 (23.6%)	60 (51.3%)	48 (65.8%)		
Mixed	10 (9.1%)	6 (5.1%)	4 (5.5%)		
**pT stage**					
T1 and T2	80 (72.7%)	76 (65.0%)	32 (43.8%)	0.002	16.573
T3	23 (20.9%)	34 (29.1%)	34 (46.6%)		
T4	7 (6.4%)	7 (6.0%)	7 (9.59%)		
**pN stage**					
N0	16 (14.5%)	13 (11.1%)	9 (12.3%)	0.033	13.753
N1	50 (45.5%)	61 (52.1%)	20 (27.4%)		
N2	25 (22.7%)	28 (23.9%)	27 (37.0%)		
N3	19 (17.3%)	15 (12.8%)	17 (23.3%)		
**pM stage**					
M0	105(95.5%)	104 (88.9%)	64 (87.7%)	4.289	0.117
M1	5 (4.5%)	13 (11.1%)	9 (12.3%)		
**AJCC stage (sixth** **edition)**					
I	16 (14.5%)	10 (8.5%)	5 (6.8%)	0.006	17.956
II	39 (35.5%)	46 (39.3%)	12 (16.4%)		
III	28 (25.5%)	36 (30.8%)	31 (42.5%)		
IV	27 (24.5%)	25 (21.4%)	25 (34.2%)		
**EBV infection**					
Negative	98 (89.1%)	97 (82.9%)	62 (84.9%)	0.087	8.141
Positive	1 (0.9%)	10 (8.5%)	7 (9.6%)		
Missing	11 (10%)	10 (8.5%)	4 (5.5%)		
**Lymphovascular invasion**					
Negative	28 (25.5%)	31 (26.5%)	14 (19.1%)	0.063	8.930
Positive	78 (70.9%)	77 (65.8%)	50 (68.5%)		
Missing	4 (3.6%)	9 (7.7%)	9 (12.3%)		
**Perineural invasion**					
Negative	59 (53.6%)	68 (58.1%)	32 (43.8%)	0.421	3.892
Positive	33 (30%)	30 (25.6%)	25 (34.2%)		
Missing	18 (16.4%)	19 (16.2%)	16 (21.9%)		
***Helicobacter pylori*** **status**					
Negative	32 (29.1%)	26 (22.2%)	14 (19.2%)	0.865	1.276
Positive	20 (18.1%)	18 (15.4%)	17 (23.3%)		
Missing	58 (52.7%)	73 (62.4%)	42 (57.5%)		

For all variables, a Chi-square test was used.

*GE* gastro‐ esophageal, *AJCC* American Joint Committee on Cancer, *EBV* Epstein-Barr virus.

**Table 2:** The clinical correlation analysis between patient characteristics and different molecular subtypes in the TCGA_STAD cohort.

Characteristics	EPC	HPC	MPC	*P* value	χ^2^/*t*
***N***	123 (33.0%)	177 (47.5%)	73 (19.6%)		
**Sex**					
Female	43 (35.0%)	59 (33.3%)	31 (42.5%)	0.383	1.918
Male	80 (65.0%)	118 (66.6%)	42 (62.5%)		
**Location of tumor**					
Antrum	45 (36.6%)	58 (32.8%)	35 (47.9%)	0.069	10.047
Body	37 (30.0%)	67 (37.9%)	24 (32.9%)		
Cardia, GE junction	33 (26.8%)	46 (26.0%)	10 (13.7%)		
Other	8 (6.5%)	6 (3.4%)	4 (5.5%)		
**Lauren type**					
Intestinal	68 (55.3%)	72 (40.7%)	21 (28.8%)	0.000	32.687
Diffuse and signet ring	13 (10.6%)	31 (17.5%)	30 (41.1%)		
Uncertain	42 (34.1%)	74 (41.8%)	22 (30.1%)		
**pT stage**					
T1	13 (10.6%)	5 (2.8%)	1 (1.4%)	0.011	13.142
T2, T3 and T4	108 (87.8%)	169 (95.5%)	69 (94.5%)		
Uncertain	2 (1.6%)	3 (1.7%)	3 (4.1%)		
**pN stage**					
N0	37 (30.0%)	52 (29.4%)	22 (30.1%)	0.539	3.115
N1, N2 and N3	78 (63.4%)	120 (67.8%)	46 (63.0%)		
Uncertain	8 (6.5%)	5 (2.8%)	5 (6.8%)		
**pM stage**					
M0	106 (86.2%)	158 (89.3%)	64 (87.7%)	0.534	3.144
M1	7 (5.7%)	12 (6.8%)	6 (8.2%)		
Uncertain	10 (8.1%)	7 (4.0%)	3 (4.1%)		
**Pathologic stage**					
I	26 (21.1%)	20 (11.3%)	7 (9.6%)	0.018	8.467
II, III and IV	88 (71.5%)	148 (83.6%)	61 (83.6%)		
Uncertain	9 (7.3%)	9 (5.1%)	5 (6.8%)		
***H. pylori*** **infection**					
Negative	62 (50.4%)	66 (37.3%)	16 (21.9%)	0.003	16.374
Positive	5 (4.1%)	7 (4.0%)	5 (6.8%)		
Uncertain	56 (45.5%)	104 (58.8%)	52 (71.2%)		
**Differentiation stage**					
G1 and G2	69 (56.1%)	64 (36.2%)	13 (17.8%)	0.000	32.766
G3	51 (41.5%)	111 (62.7%)	56 (76.7%)		
Uncertain	3 (2.4%)	2 (1.1%)	4 (5.5%)		
**Race**					
Asian	24 (19.5%)	31 (17.5%)	18 (24.7%)	0.019	15.172
White	72 (58.5%)	115 (65.0%)	51 (69.9%)		
Black	8 (6.5%)	3 (1.7%)	0 (0%)		
Uncertain	19 (15.4%)	28 (15.8%)	4 (5.5%)		

For all variables, a Chi-square test was used.

### The proportion of patients with the HPC subtype has surged in GC

**Fig. 3:**
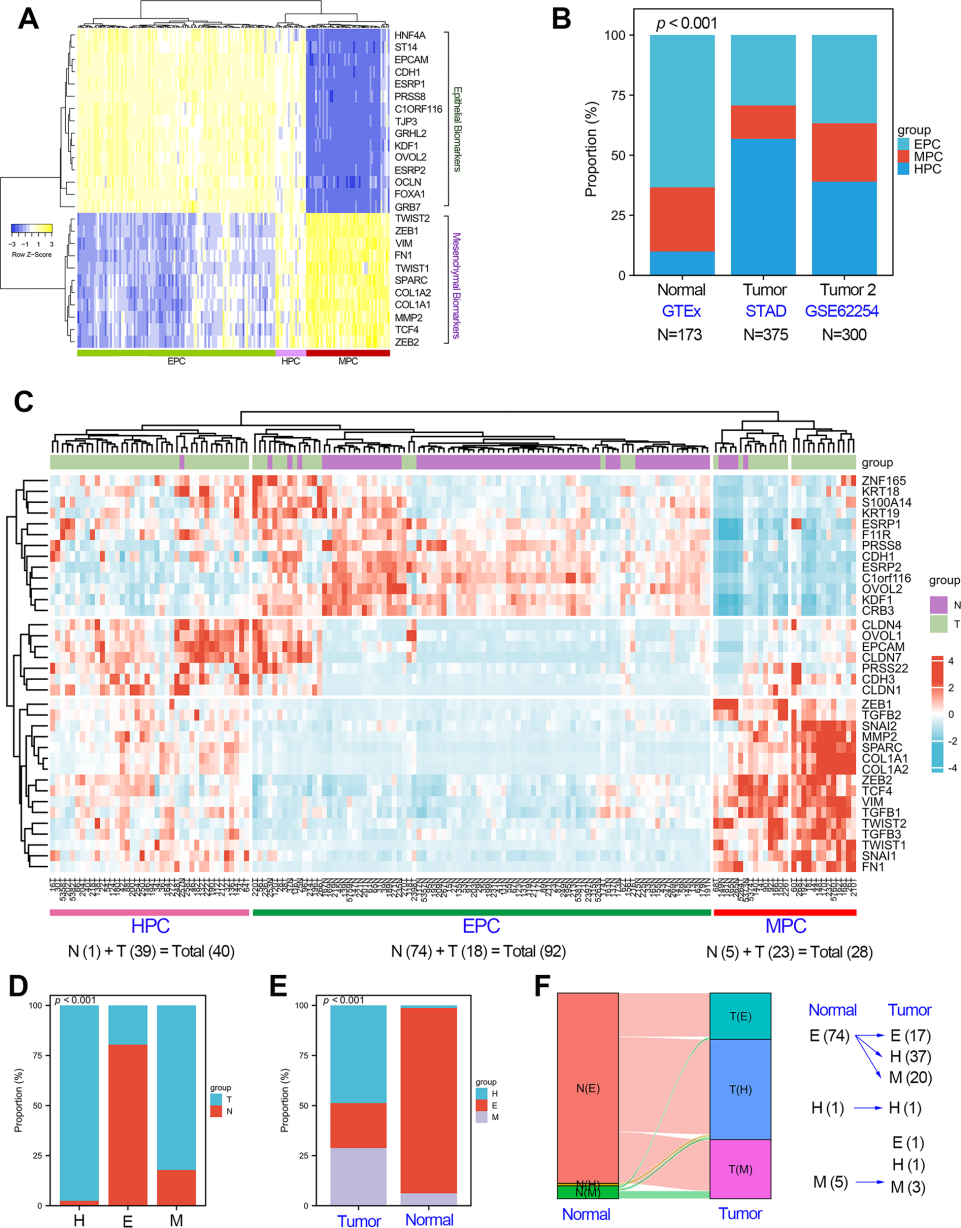
The proportion of HPC subtype increased in GC. **a** Molecular typing studies based on the expression signature of classic EMT biomarkers in normal gastric tissues (GTEx cohort). Samples from the GTEx_stomach cohort could be divided into three subtypes: EPC (*n* = 105), HPC (*n* = 53) and MPC (*n* = 17). **b** The proportion analysis of each EMT subtype in both the normal gastric cohort (GTEx_stomach) and cancerous cohorts (GSE62254 and TCGA_STAD), using a chi-square test to determine significant differences. **c** Molecular typing studies based on the expression signature of classic EMT biomarkers in the GSE122401 cohort containing 80 paired cancerous and adjacent tissues. Samples from the GSE122401 cohort could be divided into three subtypes: EPC (*n* = 92), HPC (*n* = 40) and MPC (*n* = 28). **d** The EPC subtype contains 18 cancerous samples and 74 adjacent normal samples; the HPC subtype contains 39 cancerous samples and 1 adjacent normal sample; the MPC subtype contains 23 cancerous samples and 5 adjacent normal samples. **e** The proportion analysis of each EMT subtype in both normal and tumor tissues in the GSE122401 cohort. **f** The changes of EMT subtypes corresponding to paired adjacent and cancerous tissues from the same donor. **P < 0.01.

Since EMT program is highly conserved in both normal and tumor cells, we also performed EHBMT analysis in the normal gastric cancer cohort from the GTEx database (n = 175, Fig. 3a). As expected, EHBMT also divided the normal gastric tissues into three subtypes, including EPC (n = 105, 60%), HPC (n = 17, 9.7%), and MPC (n = 53, 30.3%). The detail clustering information of per normal gastric sample was provided in Supplementary Table 3. Comparing the proportion differences of different EHBMT subtypes in GC and normal gastric, we found that the proportion of patients with the HPC subtype increased sharply in GC (Fig. 3b). On the contrary, the proportion of patients with the EPC subtype was significantly decreased in GC (Fig. 3b).

To further support the evidence that the proportion of patients with the HPC subtype increased sharply in GC, we also performed EHBMT analysis in the GSE122401 cohort containing 80 paired cancer tissues and adjacent tissues (Fig. 3c). The detailed clustering information per sample in the GSE122401 cohort is presented in Supplementary Table 4. Consistently, the GSE122401 cohort could also be divided into three subtypes: EPC (*n* = 92, 57.5%), HPC (*n* = 40, 25.0%) and MPC (*n* = 28, 17.5%). As shown in Fig. 3d, the EPC cluster contained 74 normal samples (80.4%) and 18 tumor samples (19.6%), the HPC cluster contained 39 tumor samples (97.5%) and 1 normal sample (2.5%), while the MPC cluster contained 5 normal samples (17.9%) and 23 tumor samples (82.1%). In addition, the proportion analysis of the EHBMT subtype in GSE122401 cohort suggested that the proportion of the HPC subtype was increased sharply in GC. Moreover, compared with the normal group, patients with the EPC subtype were significantly decreased as expected, while patients with the MPC subtype significantly increased, as expected, in GC (Fig. 3e). Traceability analysis shows that about 73% of EPC (*n* = 74) will be converted into HPC (n = 37) and MPC (*n* = 20) during gastric carcinogenesis (Fig. 3f).

**Fig. 4:**
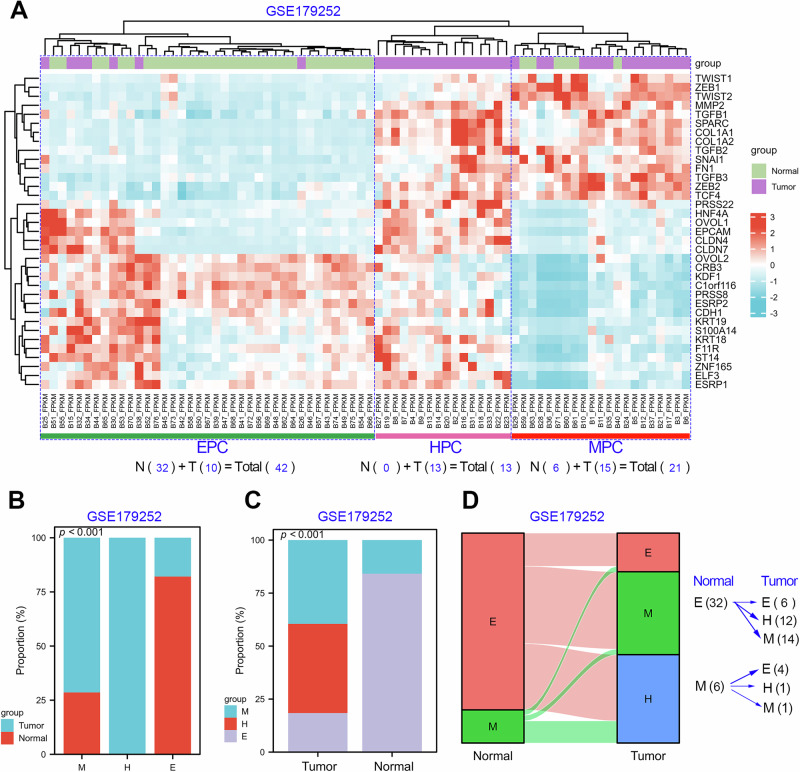
The proportion of HPC subtype was increased in the GSE179252 cohort. **a** Molecular typing studies based on the expression signature of the classic EMT biomarkers in the GSE179252 cohort containing 38 paired cancerous and adjacent tissues. Samples from the GSE179252 cohort could be divided into three subtypes: EPC (*n* = 42), HPC (*n* = 13) and MPC (*n* = 21). **b** The EPC subtype contains 10 cancerous samples and 32 adjacent normal sample; the HPC subtype contains 13 cancerous samples and 0 adjacent normal samples; the MPC subtype contains 15 cancerous samples and 6 adjacent normal sample. **c** The proportion analysis of each EMT subtype in both normal and tumor tissues in the GSE179252 cohort. **d** The changes of EMT subtypes corresponding to paired adjacent and cancerous tissues from the same donor. **P < 0.01.

In addition, we further conducted EHBMT analysis in another independent GC cohort (GSE179252) containing 38 paired cancer tissues and adjacent tissues^25^. The detailed EHBMT clustering information per sample in the GSE179252 cohort is provided in Supplementary Table 5. Similarly, the GSE179252 cohort was divided into three subtypes: EPC (*n* = 42, 55.3%), HPC (*n* = 13, 17.1%) and MPC (*n* = 21, 27.6%). As shown in Fig. 4a–c, the EPC cluster contained 32 normal samples (76.2%) and 18 tumor samples (23.8%), the HPC cluster contained 13 tumor samples (100%), while the MPC cluster contained 6 normal samples (28.6%) and 15 tumor samples (71.4%). In addition, the proportion analysis indicated that the proportion of the HPC subtype was increased sharply in the GSE179252 cohort. Moreover, we noted that patients with the normal EPC subtype were significantly decreased as expected, while patients with the MPC subtype had significantly increased as expected during GC evolution (Fig. 4c). Traceability analysis shows that 81.2% of normal EPC (*n* = 26) tissue was converted into cancerous HPC (*n* = 12) and MPC (*n* = 14) tissue during GC evolution (Fig. 4d).

### Validation of EMT heterogeneity of GC using mIHC and single-cell analysis

**Fig. 5:**
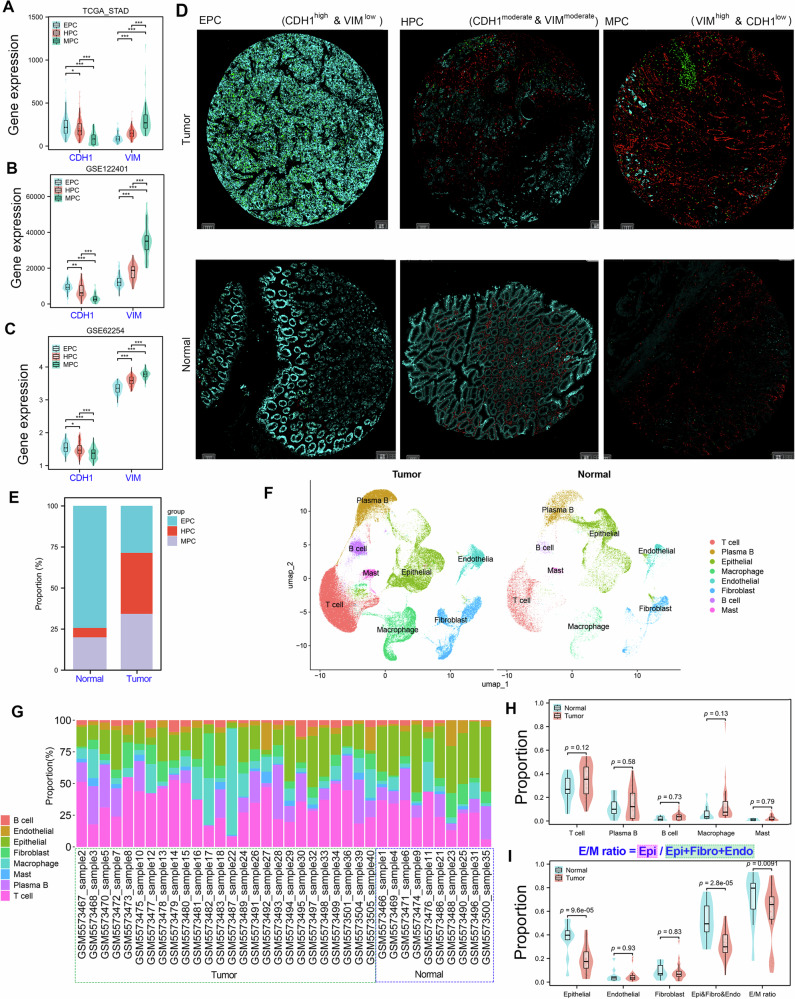
Verifying the reliability of EMT heterogeneity-based molecular typing results using mIHC method. **a**–**c** The expression levels of CDH1 and VIM in each EMT subtype from the TCGA_STAD (**a**) and GSE122401 (**b**) and GSE62254 (**c**) cohorts. **d** mIHC assay using E-cad (cyan), VIM (red) and Ki-67 (green) performed on our own GC cohort containing 35 paired adjacent normal and cancerous tissues. The tissues with high CDH1 expression and low VIM expression were considered as the EPC subtype, the tissues with low CDH1 expression and high VIM expression were considered as the MPC subtype and the tissues with moderate CDH1 and VIM expression were considered as the HPC subtype. **e** The proportion analysis of each EMT subtype identified by the mIHC assay in our own GC cohort. **f** UMAP plots of different cell types from tumor and nonmalignant normal samples of patients with GC from the GSE183904 dataset. **g** The proportion analysis of different cell types per sample from the GSE183904 dataset. **h** The immune cell type proportion analysis in the GSE183904 cohort between normal samples and tumor samples. **i** The proportion analysis of the EMT-related cell type and E/M ratio in the GSE183904 cohort between normal samples and tumor samples. E/M ratio = epithelial cells/(epithelial + fibroblast + endothelial) cells. **P < 0.01.

In EMT signaling, E-cadherin (CDH1) and VIM are classic epithelial and mesenchymal markers, respectively. As expected, gene expression analysis showed that CDH1 has the highest expression in the EPC subtype and the lowest expression in the MPC subtype of all three GC cohorts. On the contrary, VIM showed the highest expression in the MPC subtype and the lowest expression in the EPC subtype of all three GC cohorts (Fig. 5a–c). Furthermore, gene expression correlation analysis showed that the combined use of VIM and CDH1 enables effective subtype stratification of GC samples (Supplementary Fig. 1a–c). Immunohistochemistry is the gold standard for determining the reliability of molecular typing methods for tumor/normal tissues. Thus, we conducted a mIHC assay using antibodies of E-cad (cyan), VIM (red) and Ki-67 (green) in the GC cohort containing 35 paired cancerous and adjacent tissues (Fig. 5d).

Samples with high CDH1 expression and low VIM expression (ratio of CDH1/VIM ≥3) were defined as the EPC subtype, samples with low CDH1 expression and high VIM expression (ratio of CDH1/VIM ≤0.5) were defined as the MPC subtype and samples with moderate expression of CDH1 and VIM (0.5< ratio of CDH1/VIM <3) were defined as the HPC subtype (Supplementary Fig. 1d,e). The mIHC method identified 10 EPC samples, 13 HPC samples and 12 MPC samples in stomach cancer tissues and 26 EPC samples, 2 HPC samples and 7 MPC samples in adjacent tissues (Fig. 5e).

Kumar and colleagues generated a comprehensive single-cell atlas of GC comprising 31 primary gastric tumor samples representing clinical stages I–IV and histological subtypes^27^. Through reanalyzing of the single-cell dataset (GSE183904), we obtained 8 cell clusters, including T cell, plasma B, epithelial, macrophage, endothelial, fibroblast and mast cells based on the expression of known markers: CD3D (T cell), TNFRSF17 (plasma B), CDH1 (epithelial), CD163 (macrophage), PECAM1 (endothelial), FN1 (fibroblast), MS4A1 (B cell), KIT (mast) (Supplementary Fig. 2a–c).The top ten specifically expressed genes in each cell type are shown as a heat map (Supplementary Fig. 3). The quantity and clustering information per cell type in tumor and normal tissues are shown in Fig. 5f. As the number of available single cells varies across samples, we analyzed the proportion of each cell type in each sample, including 26 tumor samples and 10 normal samples (Fig. 5g). Next, we compared the proportional differences of each cell type between tumor and normal samples. As shown in Fig. 5h, the proportional differences of five immune cell types (T cells, plasma B cells, B cells, macrophages and mast cells) between tumor samples and normal samples were not significant. As expected, the proportion of epithelial cells in tumor tissues were significantly decreased (Fig. 5i). In addition, we considered the cells directly associated with the EMT signaling pathway (epithelial, fibroblast and endothelial cells) as a whole and used the E/M ratio (the proportion of epithelial cells within this group) to reflect the progression of the EMT pathway in each sample. The E/M ratio was significantly downregulated in tumors, indicating more pronounced EMT events in tumor samples (Fig. 5i). These mIHC and single-cell analysis results together confirm that visualizing tumor evolution through EHBMT is feasible.

### The molecular characteristics of different EMT subtypes of GC

**Fig. 6:**
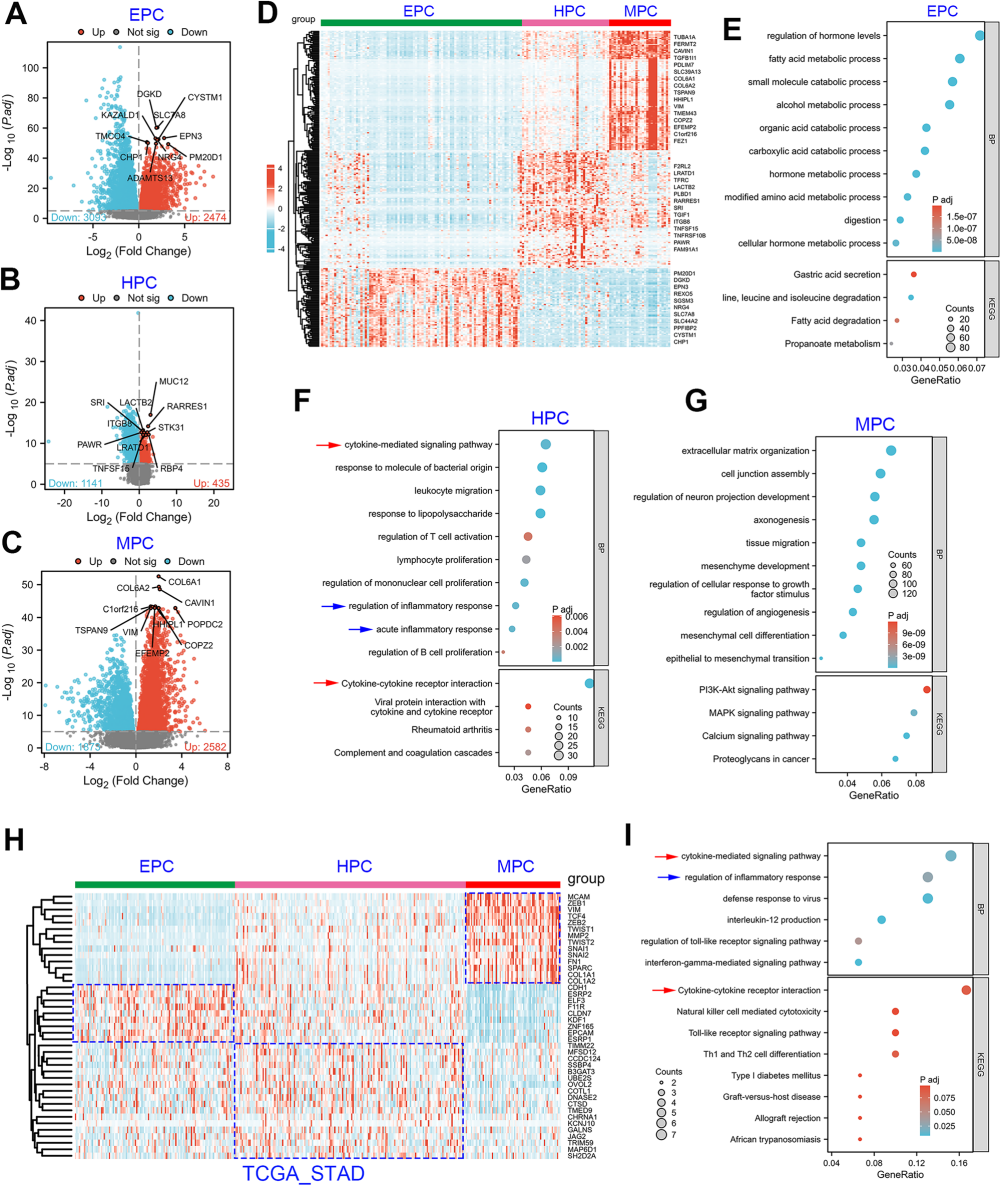
The molecular characteristics of patients with GC in each EMT subtype. **a–c** Volcano plots of the DEGs in each EMT subtype (EPC (**a**), HPC (**b**) and MPC (**c**)) from the GSE122401 cohort. The top ten upregulated genes are marked in the volcano map. **d** A heat map constructed using the genes with the most significant upregulation among different EMT subtypes in the GSE122401 cohort. **e**–**g** GO/KEGG analysis conducted using the most significantly upregulated genes of each EMT subtype (EPC (**e**), HPC (**f**) and MPC (**g**)) to reveal their representative pathways and molecular features. **h** A heat map of genes with the most significant upregulation in the HPC subtype of the TCGA-STAD cohort. **i** GO/KEGG analysis showing that the genes with the most significant upregulation in the HPC subtype of the TCGA-STAD cohort were enriched in inflammation and immune-related signaling pathways. The red arrow shows immune-related signaling pathways and the blue arrow shows inflammation-related signaling pathways.

As described above, the GSE122401 cohort consists of EPC (*n* = 92), HPC (*n* = 40) and MPC (*n* = 28) subtypes. Thus, based on the RNA-seq data of GSE122401, we conducted a strict DEG analysis (*P* < 0.0001) to identify the molecular signature for each EHBMT subtype of GC (Supplementary Tables 6–8). According to the DEG analysis, 3,093 protein-coding genes were downregulated and 2,474 protein-coding protein genes were upregulated in the EPC subtype. The top ten genes upregulated in the EPC subtype are shown in the volcano plot (Fig. 6a). Similarly, there were 435 protein-coding genes upregulated in the EPC subtype, including MUC12, RARR3S1, TNFSF15, RBP4 and so on (Fig. 6b). In the MPC subtype, 2,582 genes were significantly upregulated, including COL6A1, COL6A1, VIM and so on (Fig. 6c). Next, we performed heat map analysis of the top 100 genes upregulated per EMT subtype (Fig. 6d).

To understand the typical signaling pathways enriched in each subtype, the genes upregulated in the volcano map were used to conduct GO/KEGG analyses (Supplementary Tables 9–11). The biological process (BP) in GO analysis showed that the metabolic process of fatty acids, small molecules, alcohol, organic acid, carboxylic acid, hormones and amino acids was activated in the EPC subtype (Fig. 6e). KEGG analysis showed that the genes upregulated in the EPC subtype were enriched in the signaling pathways of gastric acid secretion, valine, leucine and isoleucine degradation, fatty acid degradation and propanoate metabolism (Fig. 6e). In the HPC subtype, significantly upregulated genes were enriched in the inflammation and immune-related signaling pathways (Fig. 6f). In the MPC subtype, significantly upregulated genes were enriched in the extracellular matrix organization, axonogenesis, neuron development, tissue migration, mesenchymal development and EMT processes (Fig. 6g). KEGG analysis showed that several well-known oncogenic signaling pathways were activated in the MPC subtype GC tissues, including PI3K–AKT, MAPK, calcium signaling and proteoglycans in cancer (Fig. 6g).

The phenomenon that HPC subtypes are closely related to inflammation and immune activation is not only present in the GSE122401 cohort but also in the TCGA-STAD cohort. We first conducted DEG analysis of the HPC subtype using RNA-seq data from the TCGA_STAD cohort. The genes significantly upregulated in the HPC subtype of the TCGA_STAD cohort are shown in a heat map (Fig. 6h). GO/KEGG analysis showed that the genes upregulated in the HPC subtype were enriched in the inflammatory response, defense response to virus, IL-12 production, toll-like receptor signaling pathway, cytokine-mediated signaling and immune-related signaling pathways (Fig. 6i).

### HPC subtype GC is associated with inflammation

**Fig. 7:**
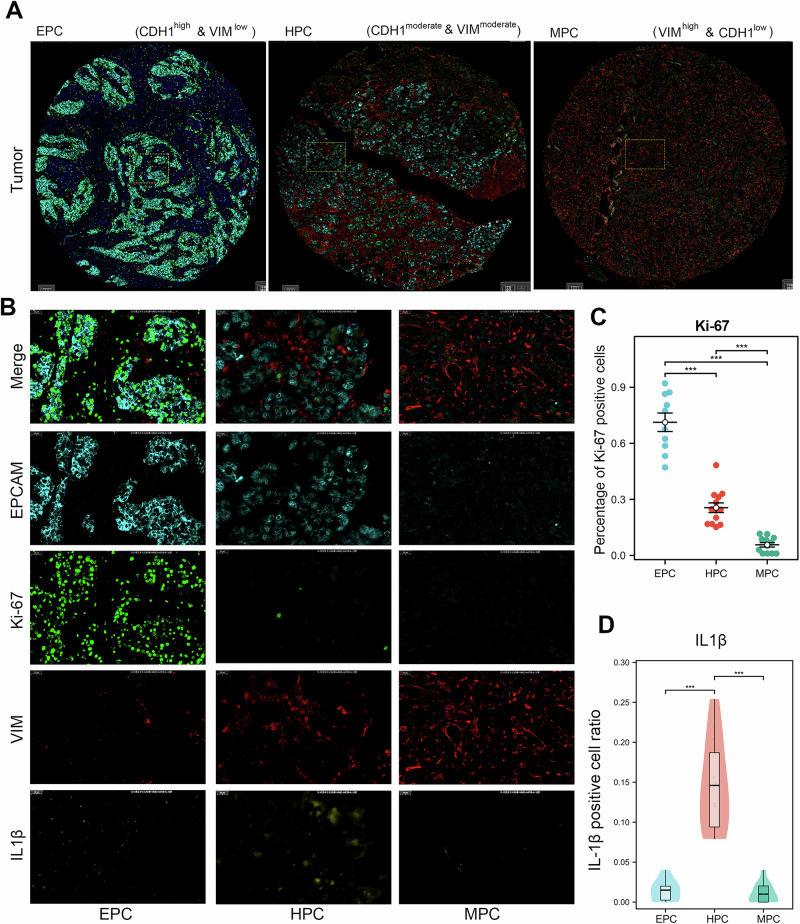
Patients with GC and the HPC subtype possess an obvious inflammatory phenotype. **a** mIHC assay using E-cad (cyan), VIM (red), Ki-67 (green) and IL-1β (yellow) performed on our own GC cohort. The representative images for EPC, HPC and MPC are shown. **b** Representative images of different EMT subtypes showing single-channel magnification. **c**, **d**, The protein expression level of Ki-67 (**c**) and IL-1β (**d**) in the different EMT subtypes from our own GC cohort. **P < 0.01.

The characteristics of EPC and MPC subtypes are well known, but little is known about the characteristics of HPC subtypes. GO/KEGG analysis showed that the HPC subtype had a significant association with inflammation (Fig. 6f). IL-1β is known to be a classic inflammation biomarker. To verify the association between the HPC subtype and inflammation, we further conducted a mIHC assay using antibodies of E-cad (cyan), VIM (red), Ki-67 (green) and IL-1β (yellow) in our own GC cohort. The corresponding representative images per EMT subtype are shown in Fig. 7a. An enlarged image of single-channel fluorescence for all three subtypes is shown in Fig. 7b. After statistical analysis, we noticed that the positivity rate of Ki-67 was highest in the EPC subtype and lowest in the MPC subtype (Fig. 7c). In addition, similar to the GO/KEGG analysis results, the HPC subtype expressed the highest level of IL-1β (Fig. 7d). These results confirm that HPC subtypes are closely related to inflammation.

### The surge in HPC subtype GC is due to an abnormal EMT signaling pathway

**Fig. 8:**
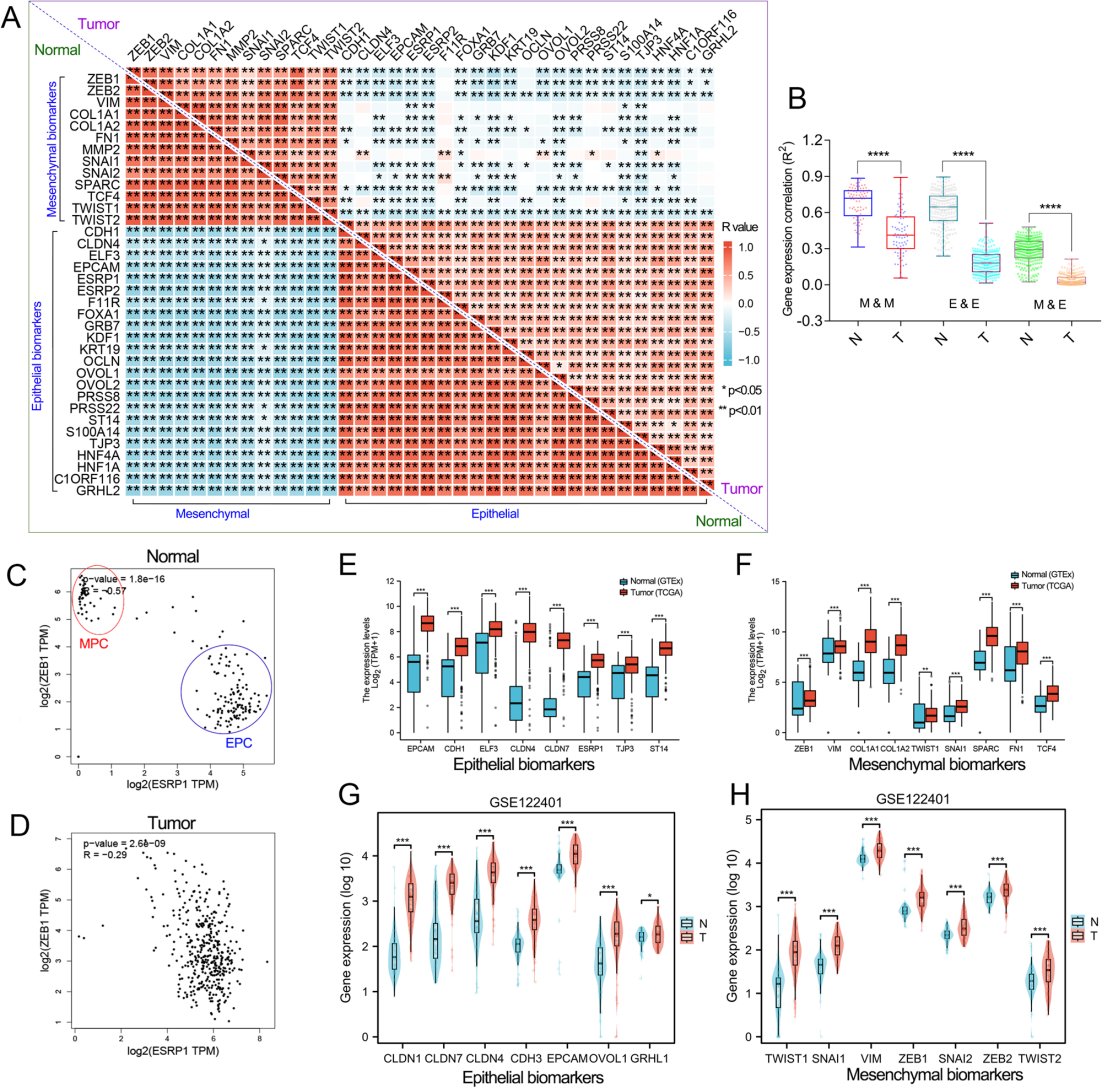
The EMT signaling pathway was dysregulated in GC. **a** Gene expression correlation among EMT-related genes was analyzed in normal stomach (GTEx cohort) and GC (TCGA_STAD) tissues. The correlation coefficient among EMT-related genes is shown in the heat map. **b** The EMT characteristics in normal gastric tissue are more pronounced than those in cancer tissue. E&E, expression correlation among epithelial genes; E&M, expression correlation between epithelial genes and mesenchymal genes; M&M, expression correlation among mesenchymal genes. **c** **d**, The gene expression correlation analysis between ESRP1 and ZEB1 performed in normal stomach (GTEx cohort; **c**) and GC (TCGA_STAD; **d**) tissues. **e**, **f** Epithelial biomarkers (EPCAM, CDH1, ELF3, CLDN4, CLDN7, ESRP1, TJP3 and ST14; **e**) and mesenchymal biomarkers (ZEB1, VIM, COL1A1, COL1A2, TWIST1, SNAI1, SPARC, FN1 and TCF4; **f**) were significantly upregulated in GC (TCGA_STAD cohort). **g**, **h** Epithelial biomarkers (CLDN1, CLDN7, CLDN4, CDH3, EPCAM, OVOL1 and GRHL1; **g**) and mesenchymal biomarkers (ZEB1, VIM, SNAI1, SNAI2, TWIST1, TWIST2 and ZEB2; **h**) were significantly upregulated in GC (GSE122401 cohort). **P < 0.01.

These results show that there is a significant difference in EMT heterogeneity between normal gastric tissues and stomach cancer tissues. Notably, a considerable portion (approximately 50%) of normal EPC subtype samples have been converted into HPC cancerous tissues, leading to a decrease in the proportion of EPC subtype and an increase in the proportion of HPC subtype in GC. However, the reasons for this difference remain unclear. EHBMT analysis showed that, compared with GC samples, normal gastric tissues had more significant EMT features. The mutually exclusive characteristics of epithelial and mesenchymal markers were very obvious in EPC and MPC subtypes (Figs. 2a, b and 3a). To further confirm this phenomenon, we conducted an expression correlation analysis between epithelial marker genes and mesenchymal marker genes in normal gastric tissue and GC tissue, respectively (Fig. 8a). The results showed that the co-expression coefficients between mesenchymal markers and epithelial markers in normal gastric tissue, as well as the negative correlation between epithelial and stromal markers, were significantly higher than those in GC tissues (Fig. 8b). Here, we took ESRP1 (epithelial biomarker) and ZEB1 (mesenchymal biomarker) as an example. In normal gastric tissues, ESRP1_high samples and ZEB1_high samples were clearly divided into two groups. However, ESRP1_high samples and ZEB1_high samples can no longer be clearly distinguished in GC tissues (Fig. 8c, d). The sharp increase in the proportion of HPC subtypes in GC was due to the abnormal EMT signaling pathway. Therefore, we conducted a gene expression analysis to determine the underlying reason why epithelial samples and mesenchymal samples cannot be clearly distinguished in GC. Gene expression analysis showed that both epithelial marker genes and mesenchymal marker genes were significantly upregulated in GC (Fig. 8e–h). In normal tissues, the trend of changes in the expression of epithelial genes and mesenchymal genes should be opposite. However, in stomach cancer, the phenomenon of mutual exclusion between epithelium and stroma is no longer obvious due to the abnormal EMT signaling.

### IL-1β promotes partial EMT of epithelial cells in GC

**Fig. 9:**
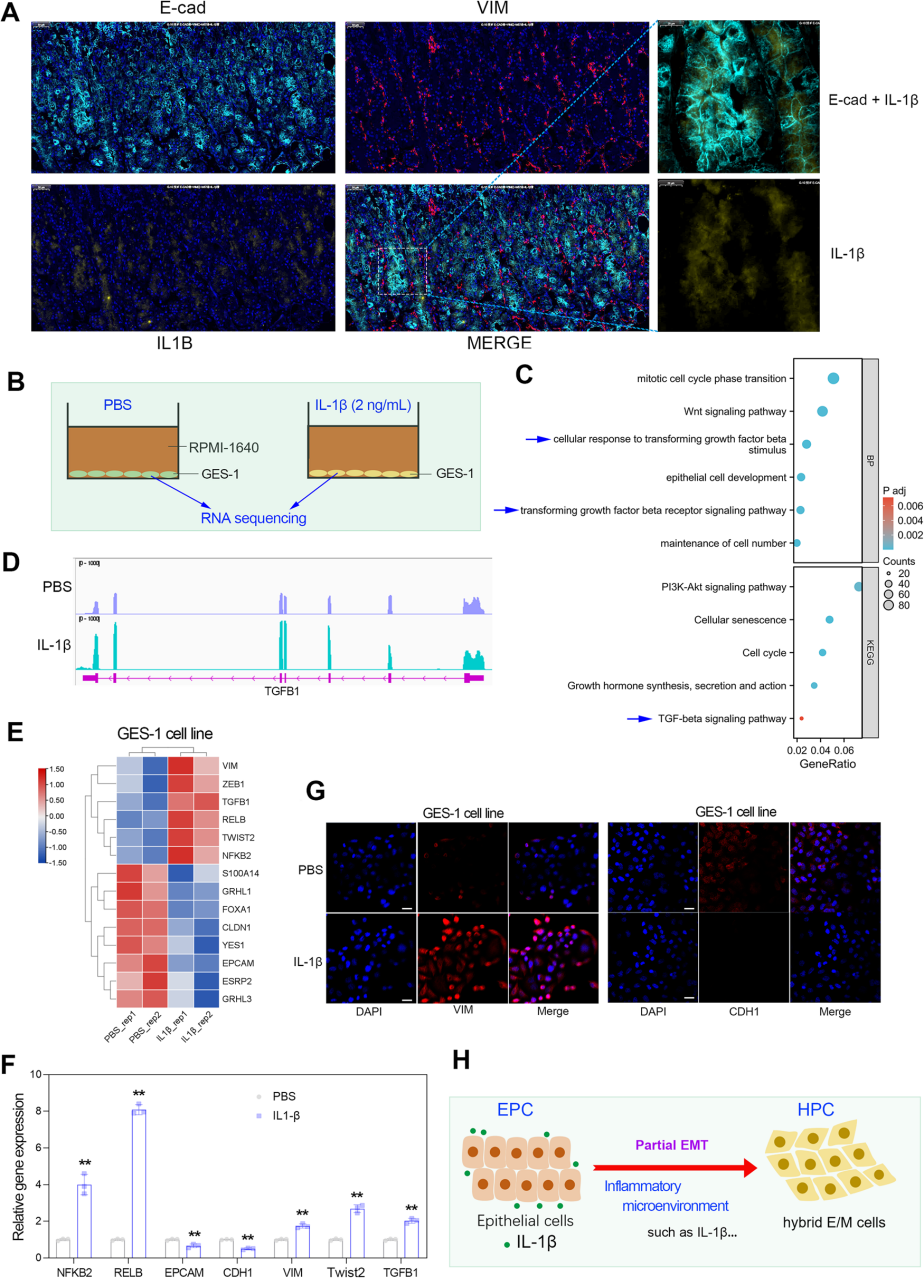
The expanding HPC subtype in GC is due to abnormal EMT signaling caused by an inflammatory microenvironment. **a** In adjacent normal gastric tissue, IL-1β was enriched in the region of epithelial cells and its surroundings. **b** The GES-1 cell line were co-incubated with recombinant human IL-1βprotein (2 ng/ml). **c** GO/KEGG analyses were conducted using the DEGs based on the RNA-seq data of recombinant human IL-1β treatment. **d**, RNA-seq analysis confirmed that recombinant human IL-1β treatment upregulated TGFB1 expression in the GES-1 cell line. **e** The differentially expressed EMT-related genes by IL-1β treatment in the GES-1 cell line. **f** mRNA expression levels of NFKB2, RELB, EPCAM, CDH1, VIM, SNAI1 and TGFB1 determined by RT–qPCR in GES-1 cells with or without IL-1β treatment. **g** Immunofluorescence demonstrates the effect of IL-1β treatment on the expression of VIM and CDH1 proteins. Scale bar, 50 μm. **h** An inflammatory microenvironment (such as IL-1β) is a key factor driving the transition from the normal EPC subtype to the cancerous HPC subtype, leading to a steep rise in the proportion of the HMT subtype from abnormal EMT signaling in GC.

We have previously demonstrated that HPC samples expressed higher levels of IL-1β (Fig. 7d). Notably, the regions expressing IL-1β clearly clearly overlap with epithelial cells (Fig. 9a). Consistently, according to the single-cell analysis of normal gastric tissues in the Human Protein Atlas (HPA) database, IL-1β is selectively expressed in gastric epithelial cells and macrophages/monocytes (Supplementary Fig. 4a,b). However, the biological function of IL-1 β expression in epithelial cells is still unclear.

To understand the biological effect of IL-1β, we selected a normal gastric epithelial cell line (GES-1) for a recombinant human IL-1β co-incubation experiment (2 ng/ml; Fig. 9b). GO/KEGG analyses based on the RNA-seq data confirmed that DEGs by IL-1β were enriched in the cell cycle, cellular senescence and TGF-β signaling pathways (Fig. 9c). Notably, RNA-seq analysis and RT–qPCR experiments confirmed that IL-1β treatment significantly upregulates the expression of NFKB2/RELB (nonclassical NF-κB signaling pathway) and activates the EMT signaling pathway in the GES-1 cell line (Fig. 9d–f). In addition, immunofluorescence experiments showed that IL-1β treatment upregulated expression of VIM and downregulated expression of CDH1 in GES-1 cells (Fig. 9g). Our previous research has shown that approximately 70–80% of normal gastric tissue EPC subtypes transform into the HPC/MPC subtypes during GC evolution (Figs. 3f and 4d). Increasing evidence has demonstrated that an inflammatory microenvironment is a decisive factor in the induction of the pathological EMT^29–31^. Therefore, our findings highlight that the inflammation microenvironment is a key factor leading to abnormal EMT signaling in GC, thereby driving the transition of the normal EPC to the cancerous HPC subtype (Fig. 9h).

## Discussion

Currently, research on Cancer Evo–Dev mainly relies on methods such as single-cell lineage tracing and clone trajectory clustering based on gene mutations^32^. However, these methods have significant drawbacks, namely high costs and strict requirements for research conditions, which have thus created a relatively high entry barrier and limited their application scope and popularity^33^. For example, single-cell lineage tracing experiments require the construction of special transgenic mouse models and proficiency in single-cell sequencing technology, as well as a high experimental cost and time^34^. The clone evolutionary trajectory analysis based on gene mutations requires in-depth sequencing of the tumor genome^35^. It is not only costly but also demands profound bioinformatics analysis skills, which significantly raises the threshold for using this method. All of these drawbacks make it difficult for ordinary laboratories to conduct research related to tumor evolution and seriously hinder the wide dissemination and application of research results. In view of this, it has become an urgent task to develop a method that is low cost, easy to operate and capable of realizing a visual analysis of tumor evolution. This is of great practical significance for promoting the in-depth development and popularization of tumor evolution research.

In the present study, we provided an EHBMT method based on bulk RNA-seq data to visualize tumor evolution and guide personalized medicine for patients with cancer. We took GC as an example to validate the reliability and feasibility of the EHBMT analysis. GC is a highly heterogeneous tumor originating from the gastric mucosal epithelial cells, which can occur in different parts of the stomach, such as the cardia-pyloric region, the fundus-cardiac region and the body of the stomach^36–38^. GC can be divided into a variety of pathologic types, such as tubular adenocarcinoma, papillary adenocarcinoma, low-adhesion carcinoma, sig-ring cell carcinoma, neuroendocrine carcinoma and hepatoid gastric carcinoma^39–41^. The heterogeneity of GC may lead to different responses to chemotherapy and targeted therapy, affecting the therapeutic effect and prognosis^42^. Therefore, a thorough understanding of the heterogeneity of GC is of great significance for the development of new diagnostic markers, therapeutic targets and personalized treatment strategies.

Clustering patients with GC using the EHBMT method is highly acceptable and feasible. First, the EHBMT method has been proven to be feasible in multiple independent GC queues. More importantly, this method has been further verified by an experimental validation of an external GC cohort using the mIHC method and single-cell analysis (Fig. 5). According to EHBMT, GC can be divided into three subtypes: EPC, HPC and MPC. Clinical analysis showed that the EPC subtype tends to be intestinal GC with shallow infiltration and a good prognosis, while the MPC subtype tends to be diffuse GC with deep infiltration and a poor prognosis (Tables 1 and 2). Consistently, Cristescu and colleagues reported that patients with GC and the EMT subtype possessed the worst clinical outcomes^19^. More importantly, the molecular subtyping results of EHBMT for four independent GC cohorts enrolled in this study were validated by the results of two different deconvolution analyses (Supplementary Fig. 5).

The biggest highlight of this study is that EHBMT can be applied into normal gastric and GC tissues to visualize cancer evolution as the cellular EMT program is highly conserved in normal/tumor cells. Previous molecular subtyping studies mostly focused on cancer tissues and lacked detail on the types of normal tissues. In this study, we conducted a proportion analysis for different EHBMT subtypes in normal gastric and GC tissues and discovered a steep rise in the proportion of the HPC subtype in GC evolution. Specifically, a traceability analysis based on the EHBMT subtypes in the GSE122401 and GSE179252 cohorts shows that gastric carcinogenesis was accompanied by an obvious transition from the normal EPC subtype to the cancerous HPC subtype.

At present, there is still a lack of sufficient understanding of the biomarkers and molecular characteristics of HPC. Thus, our next goal is to understand the molecular characteristics of HPC. The GO/KEGG analyses confirmed that inflammation and immune related pathways were active in the HPC subtype (Fig. 6). This was further verified by mIHC assays, as the inflammation factor IL-1β was highly expressed in HPC subtype GC tissues (Fig. 7d). In addition, gene expression analysis showed that HPC subtype GC tissues possessed moderate expression of epithelial and mesenchymal biomarkers (Fig. 7b). Thus, we speculated that it is the abnormal EMT signaling (simultaneous upregulation of both epithelial marker genes and mesenchymal marker genes) in GC that causes the sharp increase in the proportion of cancerous HPC subtypes (Fig. 8).

Chronic inflammation is a common driver for the occurrence and development of the vast majority of cancers, since it provides selective pressure and a microenvironment for Cancer Evo–Dev. However, the specific process and the key molecular events therein have never been fully elucidated. EHBMT analysis confirmed a surge in the proportion of the HPC subtype during GC evolution. Interestingly, a recent publication by Youssef and colleagues also found that the noninvasive p-EMT (EMT trajectory 2, called EMT-T2) in renal fibrosis or cancer is associated with inflammation^43^. Furthermore, increasing evidence has shown that an inflammatory microenvironment is a decisive factor in the induction of the pathological EMT^29–31^. EHBMT analysis and mIHC assays confirmed that the HPC subtype had obvious inflammatory characteristics and a high expression of IL-1β. EMT and inflammation signaling pathways in the epithelial cells (GES-1) exposed to IL-1β were significantly activated (Fig. 9b–f). Notably, several publications have found that IL-1β is an important mediator of the EMT process^44–47^; therefore, we speculated that an inflammatory microenvironment (such as IL-1β) might be a key driver for the transition from the normal EPC subtype to the cancerous HPC subtype during GC evolution.

Although EHBMT can reveal the contribution of the EMT signaling pathway in the process of tumor evolution, it also has some limitations. First, for nonpaired cohorts, we can only analyze the proportional changes of each subtype, and it is impossible to determine whether a specific patient is suitable for EMT-targeted therapy. For instance, patients in whom both normal and tumor tissues fall into the EPC subtype may not necessitate EMT-inhibiting therapy. Therefore, we recommend using EHBMT to visualize tumor evolution in paired tumor cohorts as the results of its traceability analysis are conducive to personalized and precise medicine for patients. Besides, since the EHBMT method relies on bulk transcriptome data, factors such as the quality of the transcriptome data and whether it has been normalized will directly affect the clustering results of samples, thereby influencing the visualization of tumor evolution. In addition, defining GC EMT subtypes solely by CDH1 and VIM expression may be inaccurate. A more precise approach would involve the combined analysis of more EMT-related markers via mIHC.

## Supplementary information

Supplementary Information

Supplementary Tables 1–11

## Data Availability

The datasets generated during the current study are available in this Article or its Supplementary Information.
