# Supplementary material for: EHBMT, a method for visualizing tumor evolution, identifies a surge in gastric cancer with hybrid epithelial–mesenchymal phenotypes due to an inflammatory microenvironment
Source: Exp Mol Med. 2025 Nov 3;57(11):2440–57. doi: 10.1038/s12276-025-01570-6 (PMC12686072; doi:10.1038/s12276-025-01570-6)
Supplement: Supplementary file 1 — Supplementary Information [file 12276_2025_1570_MOESM1_ESM.pdf]

Supplementary Information

Supplementary Table 1. The clustering information of the samples in the GSE62254 cohort.

Supplementary Table 2. The clustering information of the samples in the TCGA_STAD cohort.

Supplementary Table 3. The clustering information of the samples in the GTEx stomach cohort.

Supplementary Table 4. The clustering information of the samples in the GSE122401 cohort.

Supplementary Table 5. The clustering information of the samples in the GSE179252 cohort.

Supplementary Table 6. The differentially expressed protein-coding genes in the EPC subtype of the GSE122401 cohort.

Supplementary Table 7. The differentially expressed protein-coding genes in the HPC subtype of the GSE122401 cohort.

Supplementary Table 8. The differentially expressed protein-coding genes in the MPC subtype of the GSE122401 cohort.

Supplementary Table 9. The significantly upregulated protein-coding genes ($p<0.00001$) in the EPC subtype of the GSE122401 cohort were collected for conducting the GO/KEGG analysis.

Supplementary Table 10. The significantly upregulated protein-coding genes ($p<0.00001$) in the HPC subtype of the GSE122401 cohort were collected for conducting the GO/KEGG analysis.

Supplementary Table 11. The significantly upregulated protein-coding genes ($p<0.00001$) in the MPC subtype of the GSE122401 cohort were collected for conducting the GO/KEGG analysis.

Supplementary Fig. 1 The criteria for defining different EMT subtypes of gastric cancer by combining the expression levels of CDH1 and VIM. (**a-c**) The gene expression (FPKM) correlation analysis between CDH1 and VIM was performed in the three independent GC cohorts (GSE122401, GSE179252, and TCGA_STAD). (**d**) The protein expression (IHC score) correlation between CDH1 and VIM was analyzed based on the mIHC assays in our own GC cohort (**e**) The criteria for

defining different EMT subtypes of gastric cancer using the ratio of CDH1 to VIM expression levels. Tissues with a CDH1/VIM ratio ≥ 3 were defined as the EPC subtype, those with a ratio ≤ 0.5 as the MPC subtype, and those with a ratio between 0.5 and 3 as the HPC subtype.

Supplementary Fig. 2 The single-cell analysis of normal gastric and gastric cancer tissues based on the GSE183904 dataset. (**a**) The unified manifold approximation and projection (UMAP) of different cell types from the GSE183904 dataset. (**b**) Expression of canonical marker genes to define the major cell types. (**c**) The Umap plot of canonical marker genes in different cell types.

Supplementary Fig. 3 The heatmap displays the top 10 genes with the most significant specific expression for each cell type

Supplementary Fig. 4. The single-cell analysis of IL1B in normal gastric and gastric cancer tissues. **(a)** IL1B was selectively expressed in macrophages and epithelial cell in normal gastric tissues according to the Human Protein Atlas wet tool. (**b**) IL1B was specifically expressed in monocytes and epithelial cells in gastric tissues according to the GSE134520 dataset.

Supplementary Fig. 5 The molecular subtyping results based on the EHBMT method for different GC cohorts were validated using two distinct deconvolution analysis methods. (**a**) The xCell deconvolution method was employed to analyze the epithelial and stromal scores in each gastric cancer cohort. Differential analysis of these scores was then performed across EHBMT subtypes

within each cohort. (**b**) The ESTIMATE deconvolution method was used to assess the stromal scores in each gastric cancer cohort. Differential analysis of stromal scores was subsequently conducted among EHBMT subtypes within each cohort.

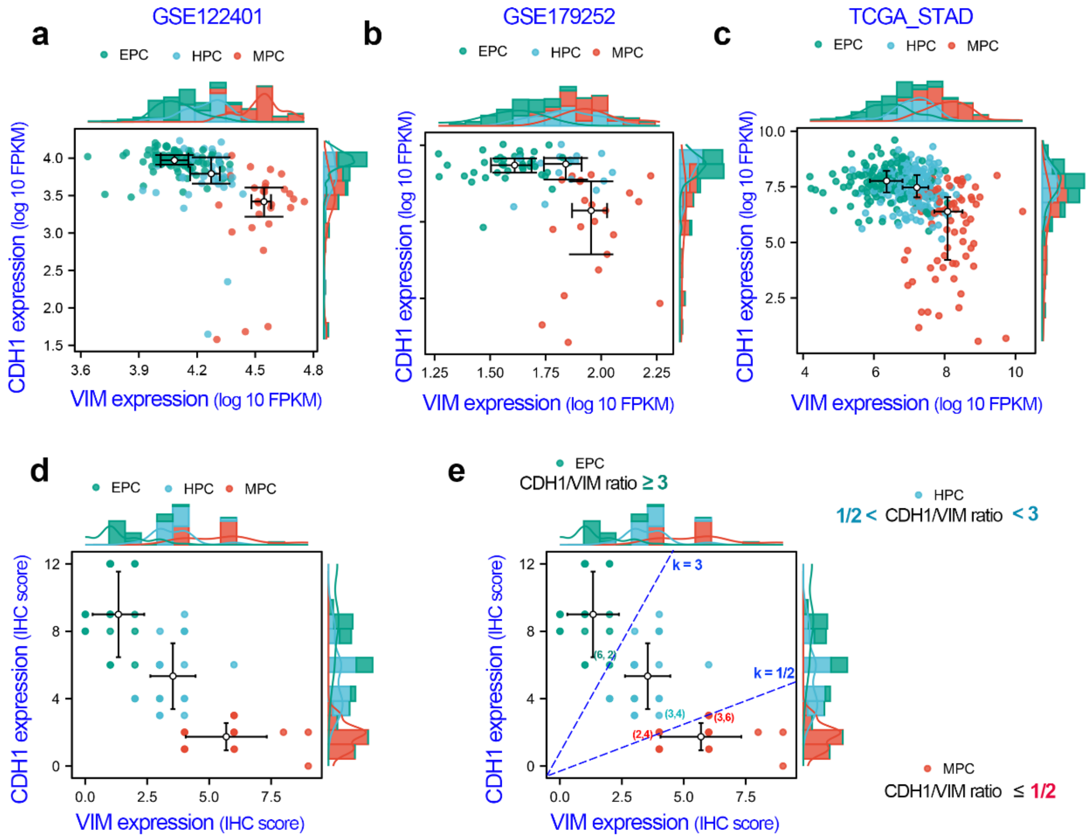

Supplementary Fig. 1

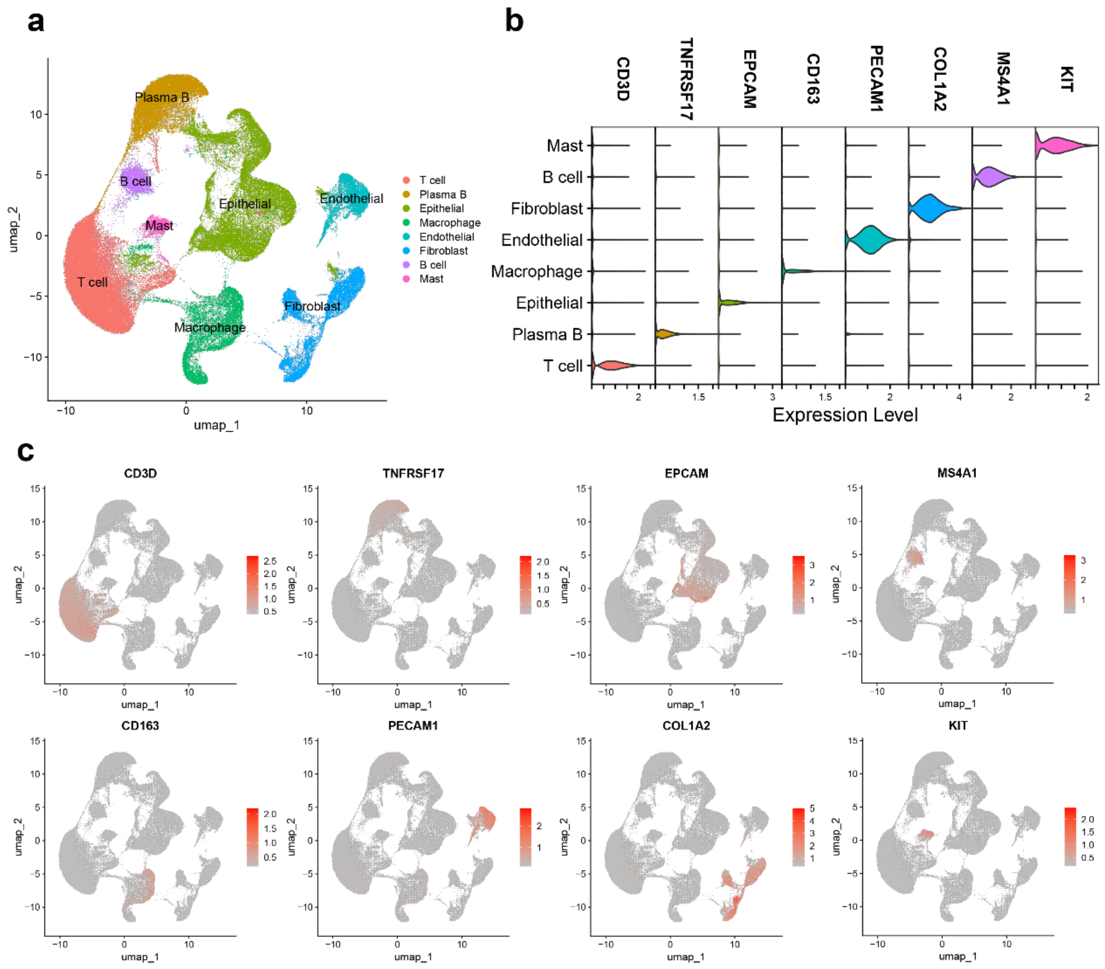

Supplementary Fig. 2

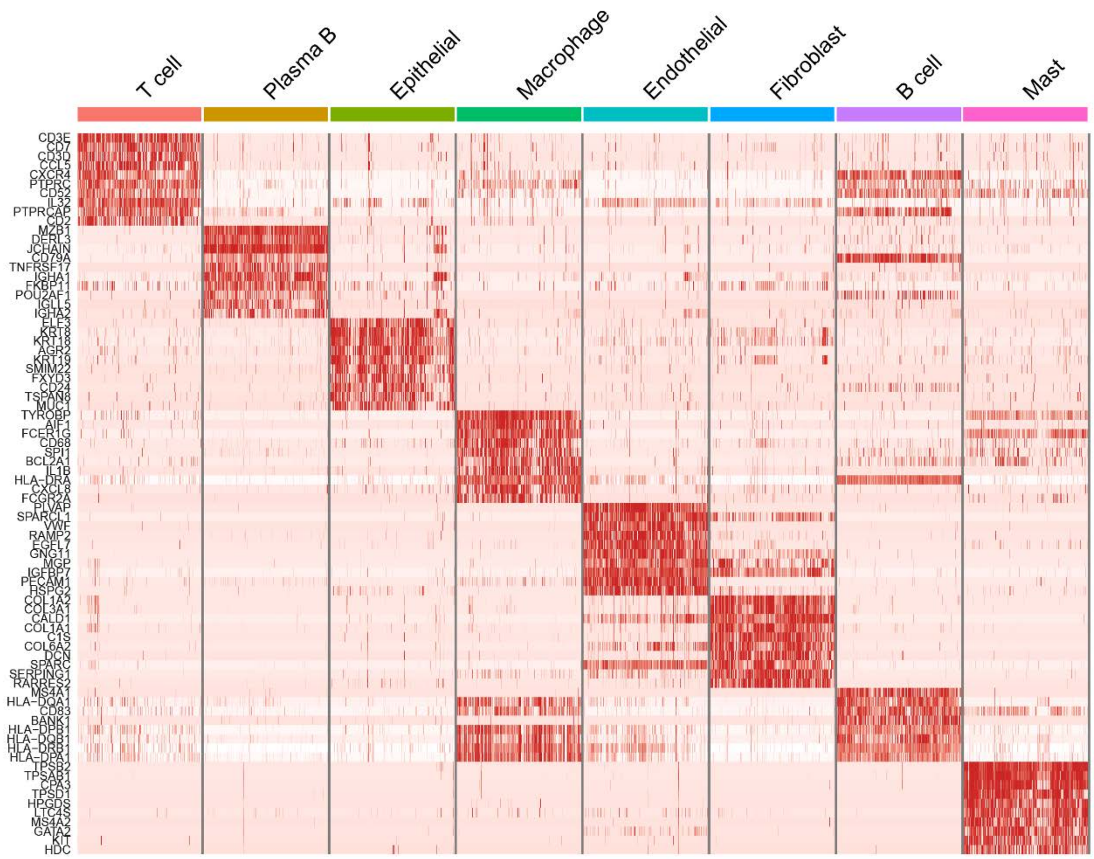

Supplementary Fig. 3

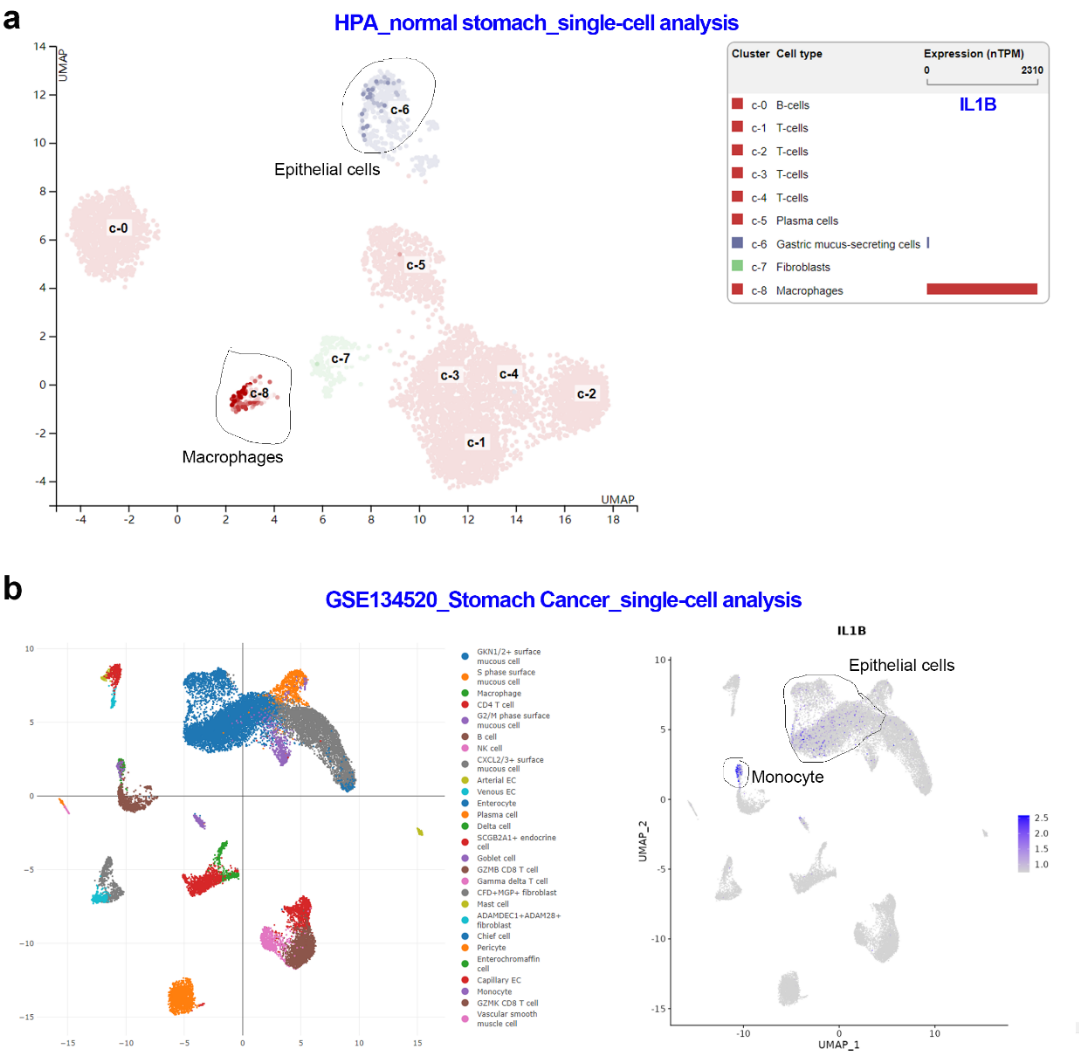

Supplementary Fig. 4

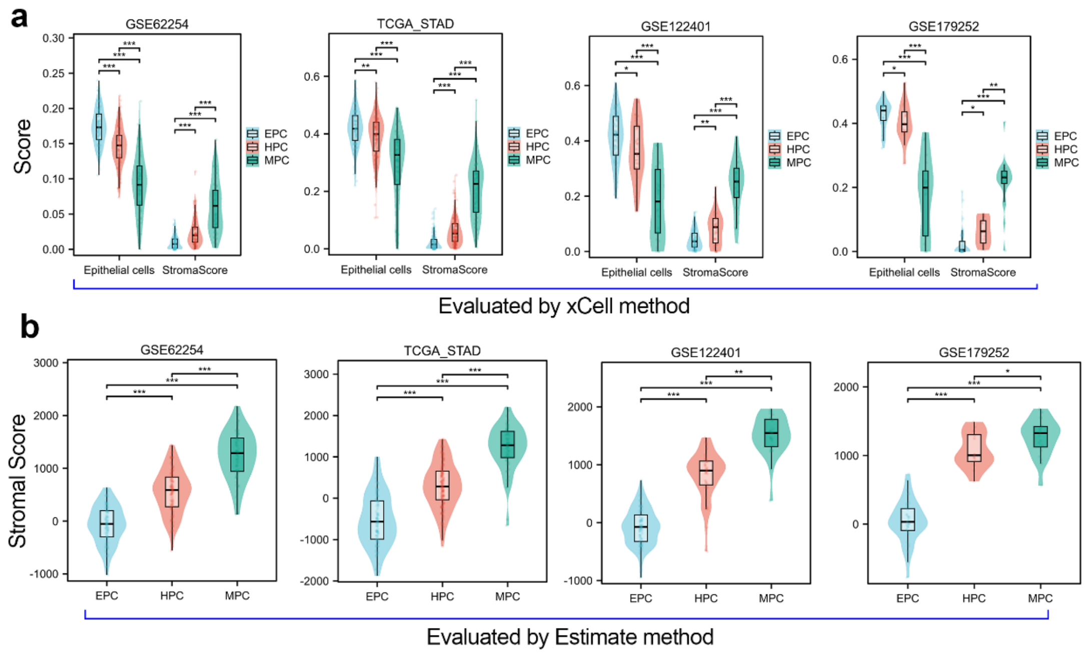

Supplementary Fig. 5
